# Adsorption of Per- and Polyfluoroalkyl Substances (PFAS) and Microcystins by Virgin and Weathered Microplastics in Freshwater Matrices

**DOI:** 10.3390/polym15183676

**Published:** 2023-09-06

**Authors:** Yucong Shi, Husein Almuhtaram, Robert C. Andrews

**Affiliations:** Department of Civil and Mineral Engineering, University of Toronto, 35 St George Street, Toronto, ON M5S 1A4, Canada

**Keywords:** LDPE, PET, PS, PMMA, PVC, drinking water

## Abstract

Microplastics and per- and polyfluoroalkyl substances (PFAS) both represent persistent groups of environmental contaminants that have been associated with human health risks. Microcystin toxins are produced and stored in the cells of cyanobacteria and may be released into sources of drinking water. Recent concerns have emerged regarding the ability of microplastics to adsorb a range of organic contaminants, including PFAS and microcystins. This study examined the adsorption of two long-chain and two short-chain PFAS, as well as two common microcystins, by both virgin and weathered microplastics in freshwater. Natural weathering of microplastic surfaces may decrease adsorption by introducing hydrophilic oxygen-containing functional groups. Up to 50% adsorption of perfluorooctanesulfonic acid (PFOS) was observed for virgin PVC compared to 38% for weathered PVC. In contrast, adsorption capacities for microcystins by virgin LDPE were approximately 5.0 µg/g whereas no adsorption was observed following weathering. These results suggest that adsorption is driven by specific polymer types and dominated by hydrophobic interactions. This is the first known study to quantify PFAS and microcystins adsorption when considering environmentally relevant concentrations as well as weathered microplastics.

## 1. Introduction

Microplastics have received recent attention with respect to their ability to adsorb various types of organic contaminants, including persistent organic pollutants (POPs) [1,2]. This property allows microplastics to serve as a transport vector for contaminants of emerging concern (CECs), which may ultimately impact human health. For example, carcinogenic polycyclic aromatic hydrocarbons (PAHs) associated with ingested microplastics have been shown to impose an incremental lifetime cancer risk (ILCR) that was 10 times higher than the recommended level of 10^−6^ [3]. As such, a need exists to better understand the adsorption behaviour of CECs by microplastics.

In addition to food and air [4], drinking water represents a direct means for microplastics to enter the human body. Microplastics have been widely observed to be present in source waters (lakes and rivers) in Europe, Asia, and North America [5,6,7]. The majority (up to 95%) of microplastics reported in freshwater are <10 μm [8,9]. Polyethylene (PE), polyethylene terephthalate (PET), and polypropylene (PP) represent over 80% of the polymers observed with approximately 70% present in the form of fragments and fibers [8,9,10,11,12]. Although drinking water treatment processes are capable of removing microplastics, reported removal efficiencies vary from 59% to >99% depending on the specific processes, raw water quality, and the size range of microplastics examined [9,11,12]. Danopoulos et al. [5] reported microplastic concentrations to range between 0.0007 and 628 particles/L when considering six different tap water studies in Europe, Asia, and North America, further highlighting their widespread presence in treated water.

Previous studies have primarily considered virgin polymers as well as marine waters when evaluating the adsorption of various POPs, including PAHs [13,14], pesticides [15,16,17,18,19], polychlorinated biphenyls (PCBs) [20,21], and per- and polyfluoroalkyl substances (PFAS) [22,23,24]. Mato et al. (2001) reported adsorbed concentrations of POPs on PP microplastics to reach 105–106 times higher than those in surrounding waters. When considering various polymer types, PE, PP, and polystyrene (PS) have shown the greatest adsorption capacity with respect to hydrophobic organic pollutants [2,15,16,24,25,26]. Depending on the specific compound, adsorption coefficient (K_d_) values may range from 3 × 10^−9^ L/µg [2] to >10^−2^ L/µg [25]. Unfortunately, similar studies that involve freshwaters are very limited in the existing literature. Organic and inorganic constituents in natural waters have been shown to impact sorption onto microplastics by competing for adsorption sites [27,28,29,30]. As such, a need exists to evaluate the adsorption of contaminants in freshwater matrices representative of drinking water sources to provide information that may be used to characterize potential health risks to consumers.

In addition to water matrix characteristics, those associated with specific microplastics may have a substantial impact on the adsorption behaviour of contaminants. Previous adsorption trials have primarily employed virgin polymers whose surface characteristics differ from those that have undergone environmental weathering [15,16,18,24,26,31,32,33,34,35]. Weathered microplastic surfaces have higher roughness and contain more oxygen functional groups than virgin microplastics, as well as higher biofilm formation potential [36,37,38,39]. Adsorption capacity has been shown to increase with surface roughness and decrease with decreasing hydrophobicity resulting from the introduction of hydrophilic oxygen-containing functional groups [39]. As such, a need exists to employ weathered microplastics in adsorption trials involving freshwaters that may serve as sources of drinking water.

PFAS represent contaminants of emerging concern with respect to both freshwater and drinking water as they have been employed in various industrial applications, including surfactants and aqueous fire-fighting foams, which has resulted in their wide distribution in the environment [40,41]. They are extremely persistent and have a strong tendency to bioaccumulate versus degrade naturally [40,42]. Removal of PFAS by conventional drinking water treatment processes is also challenging when considering the reduction of potential human health risks [43]. Epidemiological and animal studies have reported potential risks of thyroid disease, immune and reproductive system disfunction, and cancer [44]. The US Environmental Protection Agency (EPA) recently released interim health advisory values of 0.004 ng/L for perfluorooctanoic acid (PFOA) and 0.02 ng/L for perfluorooctanesulfonic acid (PFOS), two of the most common PFAS observed [45], highlighting their emerging importance. Previous studies have explored PFAS adsorption onto microplastics but have not considered weathered polymers [1,46], which are associated with different surface properties when compared to virgin polymers [28]. In addition, previous studies have focused on long-chain PFAS compounds, while a recent shift has occurred towards short-chain alternatives due to regulations limiting the use of long-chain PFAS [1,46,47]. Therefore, a need exists to assess the adsorption of both long and short-chain PFAS compounds when considering a range of virgin and weathered polymers.

In addition to anthropogenic chemical contaminants, biological toxins naturally present in source waters may also be adsorbed by microplastics and pose human health risks. Climate change has promoted the increased occurrence of algal blooms and growth of cyanobacteria [48,49]. Microcystins, especially microcystin-LR (MC-LR) and microcystin-RR (MC-RR), are among the most common cyanotoxins reported in drinking water sources [50]. They may exert severe hepatotoxic impacts on humans and animals upon ingestion [48]. Removal of microcystins can be challenging when considering conventional treatment processes, especially during cyanobacterial blooms that may dictate the use of advanced treatment technologies including activated carbon and advanced oxidation [51,52]. Guidelines and standards for maximum acceptable concentrations in drinking water for total microcystins, MC-LR, or MC-LR equivalents typically range from 1 to 1.5 µg/L across jurisdictions globally [53]. However, previous studies have employed unrealistically high concentrations of microcystins ranging from 4650 to 5000 µg/L and were limited to virgin polymers [54,55]. Thus, a need exists to assess whether adsorption of microcystins occurs when considering concentrations that may occur during cyanobacterial blooms as well as the use of weathered polymers. The primary objective of this study was to assess the adsorption of long- and short-chain PFAS compounds as well as two common microcystins, onto both virgin and weathered microplastics using contaminant concentrations representative of what may be observed in natural waters.

## 2. Materials and Methods

### 2.1. Experimental Design

Initial isotherm and kinetic trials assessed adsorption of 4 different types of PFAS (perfluorobutanoic acid (PFBA), perfluorobutanesulfonic acid (PFBS), perfluorooctanoic acid (PFOA), and perfluorooctanesulfonic acid (PFOS)) by 200 µm and 1090 µm virgin LDPE using a batch method involving an artificial freshwater (AFW) matrix, adopted from a previous study by Udenby et al. [28]. Microplastic concentrations ranging from 900 to 3600 mg/L and an adsorption period of 3 weeks were employed. A fixed microplastic concentration (3600 mg/L) along with twelve distinct adsorption periods over a total of 21 days were employed in initial kinetic trials such that the rate of adsorption as well as the time required to reach equilibrium could be determined. Subsequent trials were conducted to examine adsorption of PFAS by four additional types of virgin microplastics, including PET, PS, polymethyl methacrylate (PMMA), and PVC, wherein the microplastic concentration was fixed at 3600 mg/L and samples were analyzed following 1, 2, 3, and 4-week contact periods. Microplastics that had undergone in-lab weathering were also included to examine its impact on adsorption.

Similar to those conducted for PFAS, kinetics trials were used to assess adsorption of two microcystins (MC-LR and -RR) by the same five polymer types, both virgin and following weathering. Individual microcystins were spiked at 50 µg/L; aliquots were analyzed over 24 h due to a much shorter equilibrium time, as reported in previous studies [54,55].

### 2.2. Materials and Reagents

**Figure 1:**
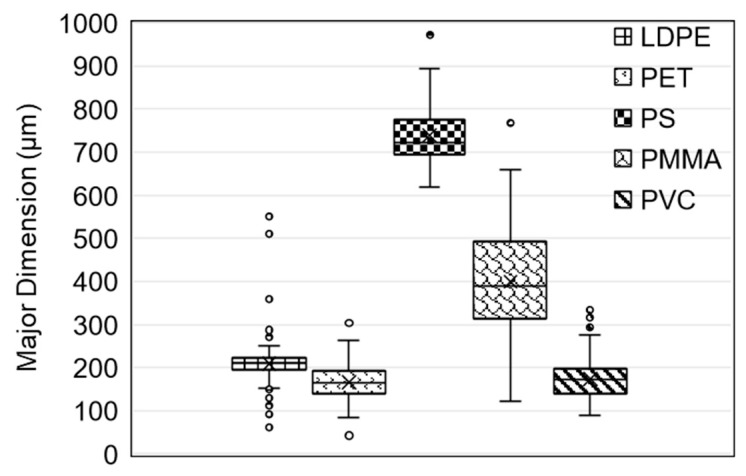
Size distribution of LDPE, PET, PS, PMMA, and PVC microspheres that were used in kinetic trials for both PFAS and microcystins. Boxes indicate quartiles, o indicates data points, and x indicates the sample mean.

Two nominal sizes (200 µm and 1090 µm) of clear LDPE microspheres obtained from Cospheric (Santa Barbara, CA, USA) were employed in initial PFAS adsorption trials. Additional LDPE, PET, PS, PMMA, and PVC microspheres that were used when examining PFAS and microcystin adsorption were obtained from Goodfellow Cambridge Ltd. (Huntingdon, UK). LDPE, PET, and PVC were sieved to obtain a size range of 125–250 µm; similarly, a size range of 300–700 µm was obtained for PS and PMMA. These ranges were selected based on availability and feasibility of use. Specific size distributions for each polymer type are summarized in Figure 1.

Individual standards for PFOA, PFOS, PFBA, and PFBS, as well as associated isotopically labelled internal standards were purchased from Wellington Laboratories (Guelph, ON, Canada). Long-chain PFOS and PFOA represent the most widely reported PFAS compounds [43], whereas PFBA and PFBS are the most abundant types of short-chain PFAS, accounting for over 50% of the total short-chain PFAS in the environment [56]. Microcystins-LR and -RR were purchased from Cayman Chemical Co. (Ann Arbor, Michigan, USA) to represent the most commonly reported microcystins [50].

All other reagents, including high-performance liquid chromatography (HPLC)-grade water, methanol, and acetonitrile that were used in LC/MS analysis, as well as sodium azide (NaN_3_), calcium chloride (CaCl_2_), potassium chloride (KCl), sodium bicarbonate (NaHCO_3_), magnesium sulfate (MgSO_4_), and nitric acid (HNO_3_) used in the preparation of AFW, were purchased from Fisher Scientific (Waltham, MA, USA).

Artificial freshwater (AFW). Preparation of AFW was adopted from a method described by Wang and Wang [57], which incorporated the addition of 1.2 mg/L potassium chloride (KCl), 58 mg/L calcium chloride (CaCl_2_), 13.0 mg/L sodium hydrogen carbonate (NaHCO_3_), 24.7 mg/L magnesium sulfate (MgSO_4_), and 25 mg/L sodium azide (NaN_3_) as a bio-inhibitor to reagent grade water (18.2 MΩ∙cm). pH of the AFW was adjusted to 7 ± 0.2 with 0.1 M nitric acid (HNO_3_). This water matrix was specifically selected so that results could potentially be compared to those by other researchers.

### 2.3. Microplastic Weathering

The microplastic weathering system employed in this study was adopted from a design described by Andrade et al. [58]. They reviewed a wide range of artificial weathering methods and proposed one that incorporates both hydrolytic and photooxidative weathering to be representative of natural weathering. The system was subsequently modified to mimic weathering conditions representative of North American freshwaters. Briefly, individual 1 L borosilicate glass cylinders were filled with approximately 20 g of microplastics, 750 mL of Elix^®^ water, and 100 mL of siliceous sand. Constant agitation and aeration were achieved by providing diffused aeration. A metal halide lamp (Daylight Blue 600W MH, Hortilux, Mentor, OH, USA) was used to simulate sunlight. Lamp output was adjusted using a dimmable ballast such that the water received radiation exposure representative of in situ conditions in North America. All polymer types examined were subjected to the same weathering process for a period of 8 weeks.

**Figure 2:**
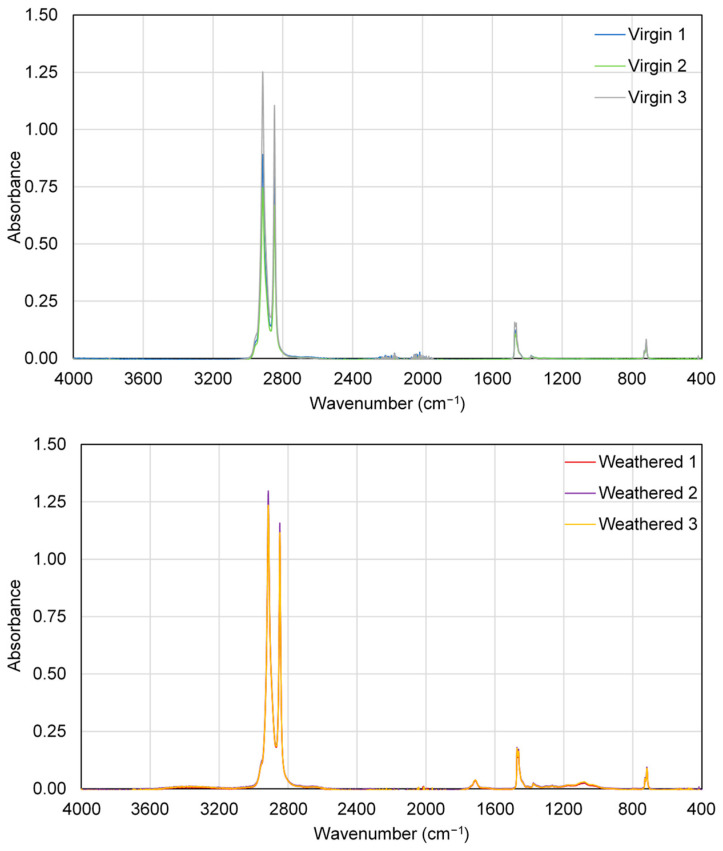
FTIR spectra for virgin and weathered LDPE (measured in triplicate).

Both virgin and weathered LDPE were analyzed using Fourier transform infrared (FTIR) spectroscopy (Thermo Scientific iS50, Waltham, MA, USA) equipped with attenuated total reflection (ATR) to characterize the impact of weathering on microplastic surfaces. Triplicate measurements were obtained and peak differences between virgin and weathered LDPE were evaluated (Figure 2). Weathered LDPE showed new peaks at 1750–1690 cm^−1^ and 1140–940 cm^−1^, corresponding to carbonyl groups (C=O) and carbon–oxygen bonds (C-O), respectively [28,58,59]. No apparent changes were observed in other portions of the spectrum. The weathering process used in this study has been shown to result in an increase in hydroxyl groups, C=O double bonds, C=O ketones, carboxylic acids, and C-O bonds for a range of polymer types in addition to LDPE [58]. These changes indicate that weathering may introduce oxygen-containing groups to microplastic surfaces, which could decrease surface hydrophobicity and potentially result in a decrease in adsorption [60].

### 2.4. PFAS Adsorption Trials

Adsorption trials for PFAS were conducted in 250 mL amber glass bottles, each filled with 245 mL of AFW to leave 5 mL of head space to ensure appropriate mixing when rotated end-over-end. Individual PFAS compounds were added using a syringe to obtain a final concentration of 500 ng/L, representative of the potential input of a wastewater effluent to a source water [61,62]. Finally, samples were spiked with 200 µm or 1090 µm virgin or weathered PE microplastics.

Six virgin and weathered microplastic doses were applied for isotherm trials (900, 1200, 1500, 1800, 2100, 2700, and 3600 mg/L), whereas the dose was fixed at 3600 mg/L for preceding kinetic trials. “Blank” AFW samples were used to evaluate the presence of any background PFAS; “control” samples containing AFW and PFAS (in the absence of microplastics) were employed to account for any potential PFAS adsorption onto the walls of glass bottles. All samples were mixed by rotating end-over-end for up to 28 days at room temperature (21.5 ± 3 °C). Initial isotherm trials were based on 21 days to reach equilibrium and analyzed at 0, 6, 18, 24, 48 and 72 h, as well as 4, 7, 11, 14, 18, and 21 d. For kinetic trials, samples were analyzed at 0, 7, 14, 21, and 28 d since minimal adsorption was observed for periods of less than 7 d. Samples were filtered by passing the water through a 45-µm stainless-steel sieve to remove microplastics followed by solid phase extraction (SPE). Internal standards were added prior to analysis using liquid chromatography with tandem mass spectrometry (LC-MS/MS).

### 2.5. Microcystin Adsorption Trials

Microcystin trials followed similar methods as described for PFAS trials. Sample volumes of 20 mL were prepared in 23 mL amber glass vials. Individual microcystins were spiked at 50 µg/L with a microplastic concentration of 3600 mg/L. Samples were then mixed end-over-end at room temperature. As adsorption of microcystins has been reported to reach equilibrium within 12 h [54,55], samples were analyzed at 0, 3, 6, 9, 12, and 24 h. Prior to analysis using LC-MS/MS, samples were passed through 0.45 µm mixed cellulose ester (MCE) syringe filters to remove microplastics.

### 2.6. Analytical Methods

LC-MS/MS methods were based on EPA Standards 537 and 533 for PFAS [63,64], and EPA Method 544 for microcystins [65]. PFAS samples were first extracted and concentrated using weak anion exchange (WAX) solid phase extraction cartridges (Waters, Mississauga, ON, Canada), while microcystin samples were analyzed using direct injection. The LC-MS/MS system included an Agilent Poroshell EC-C18 column (Agilent, Santa Clara, CA, USA). Mobile phases consisted of water and acetonitrile, which both contained 0.1% acetic acid (for PFAS) or 0.1% formic acid (for microcystins), were applied under gradient conditions. Injection volumes were 100 μL for PFAS and 40 μL for microcystins. Sample run times were 8 min for PFAS and 9 min for microcystins at a flowrate of 0.3 mL/min. The LC system was coupled to an Agilent 6460 Triple Quadrupole Mass Spectrometer system operating in electrospray ionization negative (ESI−) mode for PFAS or electrospray ionization positive (ESI+) mode for microcystins, incorporating multiple reaction monitoring (MRM). For PFAS, 13C4-PFOA, 13C4-PFOS, 13C4-PFBA, and 13C4-PFBS were used as internal standards to monitor the relative response and quantify analytes.

### 2.7. Surface Roughness

Surface roughness of microplastics was calculated as the mean surface deviation in the z-axis as measured using a KLA Tencor P16A Stylus Profilometer (Milpitas, CA, USA). To avoid the interference by particle shape, a 500 nm linear section from the scan was selected and fit to a line that is used as the z-axis. Mean surface roughness (Table 1) was calculated as the average of deviation (in absolute value) from the z-axis at each point.

### 2.8. Data Analysis

For kinetic trials, PFAS adsorption (% Adsorption) by microplastics was calculated, as well as the adsorption coefficient K_d_ at equilibrium for each PFAS compound, polymer type and weathering condition using Equations (1) and (2), respectively [23].

(1) $$\%\;\mathrm{Adsorption}=\left(1-\frac{C_{t}}{C_{0}}\right)\times 100\%$$

where $C_{0}$ and $C_{t}$ represent concentrations prior to and following adsorption.

(2) $$K_{d}=\frac{{\%\;\mathrm{Adsorption}}_{\mathrm{eq}.}}{100\%-{\;\%\;\mathrm{Adsorption}}_{\mathrm{eq}.}}\cdot \frac{V_{0}}{m_{\mathrm{sorbent}}}\;\left(\frac{\mathrm{mL}}{g}\right)$$

where $\%\;{\mathrm{Adsorption}}_{\mathrm{eq}.}$ represents the percentage absorbed at equilibrium; $V_{0}$ represents sample volume; and $m_{\mathrm{sorbent}}$ represents the mass of microplastics. When considering isotherm trials, adsorption per mass of microspheres at equilibrium ($q_{e}$) was calculated using Equation (3):

(3) $$q_{e}=\left(C_{0}-C_{e}\right)\cdot \frac{V_{0}}{m_{\mathrm{sorbent}}}$$

where $C_{e}$ represents the concentration at equilibrium.

## 3. Results and Discussion

### 3.1. PFAS Isotherm and Kinetic Trials Using 200 and 1090 µm Virgin LDPE

**Figure 3:**
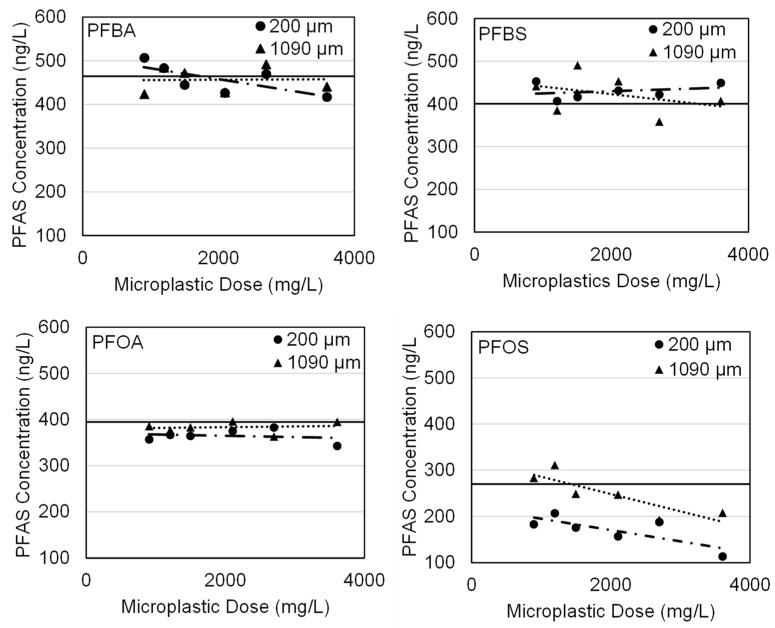
Residual PFAS concentrations in samples containing microplastics following 21 days (initial isotherm trials). Solid line represents the PFAS concentration in a control sample.

No significant adsorption by virgin LDPE was observed for any of the four PFAS compounds (Figure 3). As a result, additional trials were conducted using a range of virgin and weathered polymers.

### 3.2. PFAS Trials Using Virgin 125–250 µm LDPE, PET, and PVC and 300–700 µm PS and PMMA

**Figure 4:**
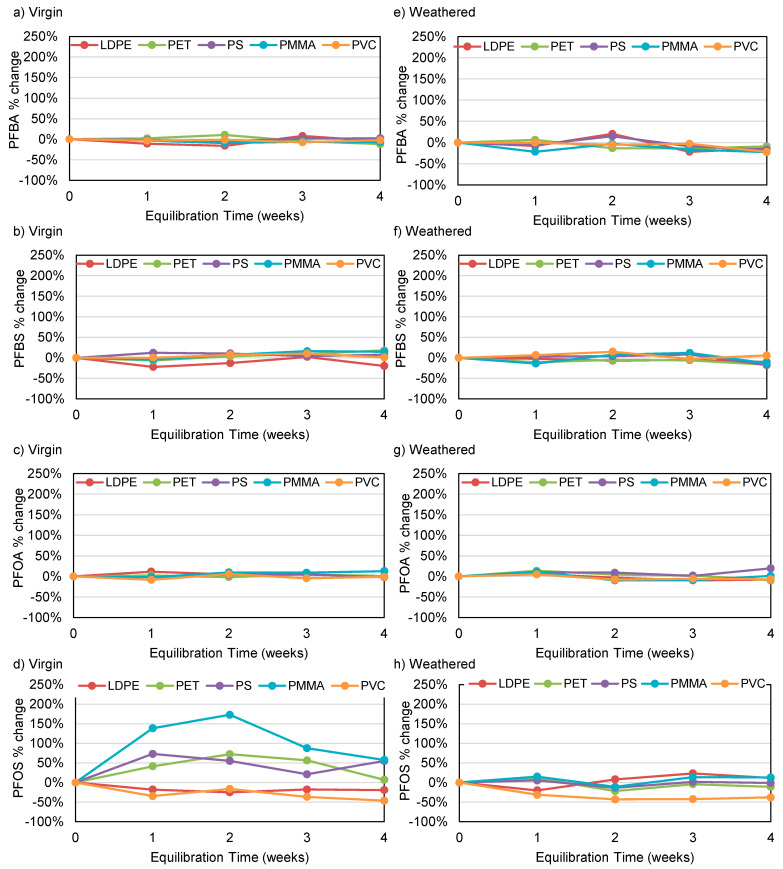
Change in residual PFAS concentration normalized to control samples without microplastics for (**a**–**d**) virgin, and (**e**–**h**) weathered microplastics.

Kinetic trials were conducted using 500 ng/L of individual PFAS compounds and 3600 mg/L of five different types of microplastics, including LDPE, PET, PS, PMMA, and PVC. Samples containing specific polymer and water were continuously mixed for up to 4 weeks and analyzed on a weekly basis to determine residual PFAS concentrations. Minor changes (<20% difference) were observed for PFBA, PFBS, and PFOA when considering all polymer types (Figure 4a–d). A 46% reduction in residual PFOS concentration by virgin PVC and a 20% reduction by virgin LDPE were observed after 4 weeks, indicating that these polymer types may adsorb PFOS in the environment.

Residual concentration data were assessed using a one-tailed paired t-test to determine if any given polymer type caused a significant decrease in PFAS concentration when compared to the control samples, which did not contain microplastics. When considering PFBA, PFBS, and PFOA, residual concentrations were not observed to significantly decrease (*p* ≥ 0.05), suggesting little to no adsorption by virgin LDPE, PET, PS, PMMA, or PVC (Figure 4a–d). In contrast, PFOS concentrations significantly decreased (adsorbed) by 25% and 46% for virgin LDPE and PVC (when compared to controls). Significant increases in PFOS were observed for virgin PET, PS, and PMMA, suggesting that this compound may have been added during polymer manufacturing and subsequently leached during trials (Figure 4d). Corresponding increases in concentration when considering weathered polymers were not observed, possibly due to leaching during weathering (Figure 4h).

**Figure 5:**
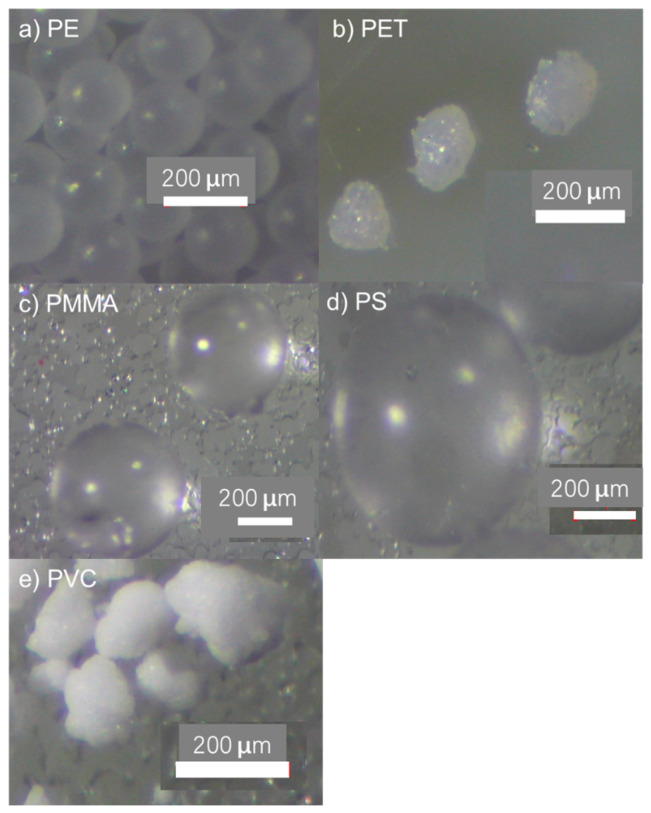
Microscope images of virgin polymers used in this study.

PFOS exhibited the highest hydrophobicity among the four PFAS analogues, while PVC had one of the roughest surfaces when examined using microscopy (Figure 5) and surface profilometry (Table 1). The fact that this combination resulted in the largest decrease in analyte concentration (Figure 4d and h) supports the hypothesis that adsorption of PFAS by microplastics increases with surface roughness as well as hydrophobicity. Therefore, it is anticipated that polymers with rough and hydrophobic surfaces will preferentially adsorb PFOS when compared to smoother, less hydrophobic polymer types.

### 3.3. PFAS Trials Using Weathered LDPE, PET, PS, PMMA, and PVC

When considering PFBA, PFBS, and PFOA, residual concentrations did not significantly differ (*p* > 0.05) from those present in control samples, suggesting that adsorption by weathered PET, PS, PMMA, and PVC microplastics is insignificant (Figure 4e–g). When considering PFOS (Figure 4h), weathered PVC caused a significant decrease (38% after 4 weeks), again suggesting significant adsorption, similar to that observed for virgin PVC (Figure 4d). In contrast to the adsorption observed for virgin LDPE, no similar impact was observed following weathering, suggesting that weathering may inhibit adsorption due to the formation of oxygen-containing surface groups that reduce surface hydrophobicity [60]. These findings suggest that the potential for adsorption of PFAS by environmental microplastics may be less than previously reported in studies that employed only virgin polymers [1,46].

### 3.4. Microcystin Kinetic Trials Using Virgin LDPE, PET, PS, PMMA, and PVC

**Figure 6:**
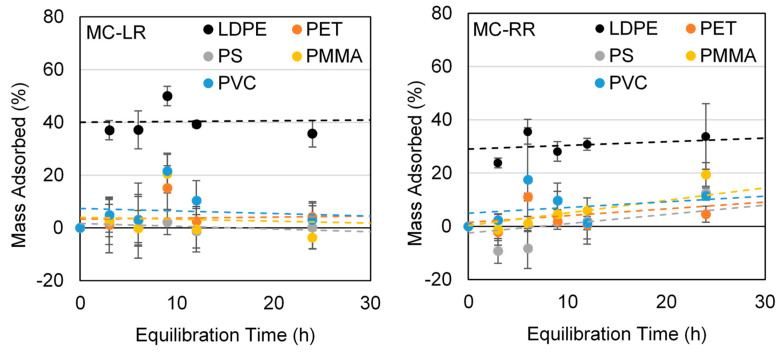
Mass of MC-LR and -RR absorbed (%) for virgin microplastics (normalized to control samples without microplastics). Vertical bars represent ± one standard deviation for triplicate samples.

Similar to PFAS trials, in order to account for changes in concentration, the mass of microcystins adsorbed was normalized to the control samples (Figure 6) that did not contain microplastics. The 95% confidence intervals were calculated based on triplicate samples using the Fieller Method. When considering all polymer types, only LDPE resulted in significant adsorption for both MC-LR and -RR (*p* < 0.01), reaching equilibrium within 24 h. Approximately 40% and 36% adsorption were observed for MC-LR and -RR, respectively. For the remaining polymer types, residual concentrations were similar to those of the controls, except for PMMA (*p* = 0.04) and PVC (*p* = 0.01) where potential adsorption of MC-RR was observed to be much less than for LDPE. As the trials were conducted at pH 7.0, the two microcystin analogues exhibited similar hydrophobicity [66]. As such, it is reasonable to assume that they would have similar adsorption characteristics. Although PMMA and PVC showed potential adsorption of MC-RR (*p* < 0.05), it was less substantial when compared to LDPE, further confirming LDPE to have the highest affinity for microcystin adsorption of any polymer examined.

Adsorption capacities of MC-LR and -RR by virgin LDPE microplastics were calculated to be 5.14 and 4.86 µg/g, respectively, similar to the values reported by Moura et al. [54] for the adsorption of MC-LR by 100 µm PP microplastics (<5 µg/g). The same authors further suggested that smaller particle sizes (15–25 µm), as well as more hydrophobic microcystin analogues (-LW and -LF), could increase adsorption significantly. When considering potential health concerns arising from the interaction of microcystins and microplastics, it should be noted that smaller microplastics <10 µm represent the most abundant size range in the environment [8], and are capable of adsorbing a greater amount of microcystin per unit mass when compared to the larger microplastics employed in the current study.

Differences observed with respect to adsorption may be attributed in part to the physical properties of the polymers that were considered. LDPE represents a rubbery or amorphous polymer, whereas the other four types may be deemed as glassy or crystalline [67]. Rubbery polymers encourage diffusion [67], which could explain the higher adsorption of microcystins by LDPE. Similarly, Moura et al. [54] examined the adsorption of microcystins in freshwater and reported the more rubbery virgin PP to have higher adsorption affinity when compared to glassy PET. Findings from the current work and previous studies suggest the primary adsorption mechanism is likely multilayer adsorption whereby surface interactions as well as diffusion occur within rubbery polymers such as polyethylene.

### 3.5. Microcystin Trials Using Weathered PET, PS, PMMA, and PVC

**Figure 7:**
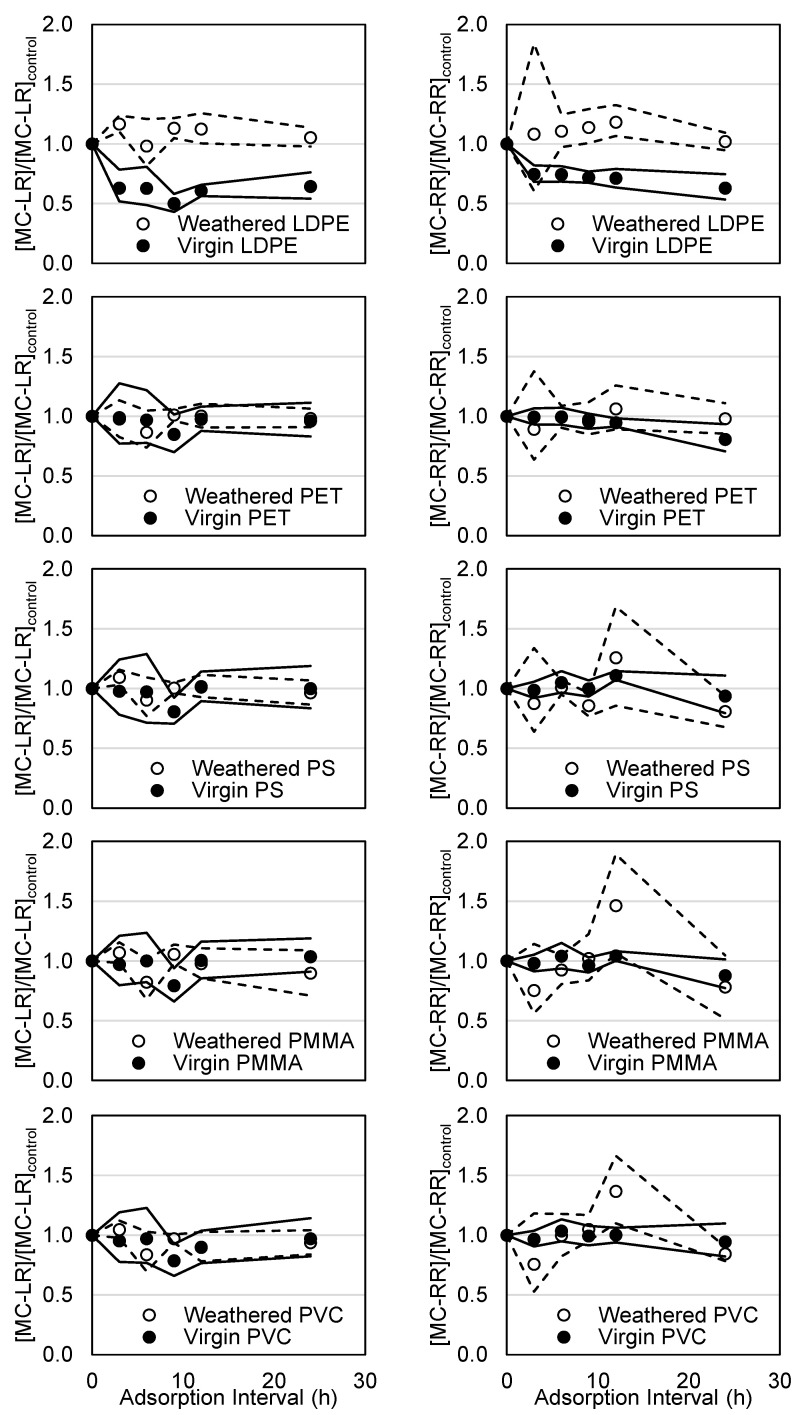
Residual MC-LR and -RR concentrations (normalized to control samples) for virgin and weathered LDPE, PET, PS, PMMA, and PVC. Horizontal lines represent upper and lower bands of 95% confidence intervals for virgin (solid) and weathered (dashed) microplastics.

Adsorption of MC-LR and -RR was quantified using weathered LDPE, PET, PS, PMMA, and PVC under the same experimental conditions that were used for virgin polymers (Figure 7). When considering all weathered polymer types, observed residual concentrations did not significantly differ from the controls (*p* > 0.05), suggesting minimal adsorption. A similar conclusion can be drawn when values are compared on a percent basis as all remained within 10% of the controls. It is notable that the significant adsorption of both MC-LR and -RR by virgin LDPE was not observed following weathering.

Weathering has been reported to increase oxygen functional groups on microplastic surfaces, which increase surface hydrophilicity and decrease adsorption affinity [36,37,38,39]. Other impacts have also been reported in the literature. Ding et al. [68] examined the adsorption of PAHs by 1 µm virgin as well as thermally weathered PS (75 °C in freshwater and seawater for 1–3 months) in ultrapure water. The authors reported weathering to significantly (90% C.I.) decrease adsorption of PAHs due to increased oxygen-containing surface groups that may form hydrogen bonds with surrounding water molecules, despite a small particle size (1 µm). Hataley [69] conducted batch adsorption trials using virgin and naturally weathered polymers (in lake water for 10 weeks), which included LDPE, PET, PS, and PVC ranging from 3–5 mm in size. The authors reported weathering to decrease adsorption of microcystins in ultrapure water. In contrast, they suggested in situ weathering to enhance adsorption of microcystins for all polymer types. This may potentially be explained by increased surface roughness associated with weathering that encourages adsorption [36,37,38,39]. Few studies have considered this factor when characterizing weathered microplastics and laboratory weathering methods (Table S1).

Neither Ding et al. [68] nor the current study considered biofilm formation in the weathering methodology. Ding et al. [68] applied thermal heating at 75 °C, whereas the current study employed UV irradiation as well as abrasion by sand particles as described by Andrade et al. [58] to represent environmental weathering. Although Hataley [69] employed microplastics exposed to lake water prior to lab trials, the conditions were less eutrophic than the lake used for in situ trials. As such, they reported significant adsorption of microcystins by weathered microplastics despite using large particle sizes (3–5 mm), suggesting that adsorption of microcystins by microplastics in natural waters may be significant despite minimal adsorption observed in the laboratory.

## 4. Conclusions

Four PFAS compounds (PFBA, PFBS, PFOA, and PFOS) as well as two microcystin congeners (MC-LR and -RR) were examined with respect to adsorption by five different types of both virgin and weathered microplastics (LDPE, PET, PS, PMMA, and PVC). Adsorption was observed for PFOS by virgin PVC and LDPE as well as MC-LR and -RR by virgin LDPE. Weathering resulted in a decrease in the adsorption of PFOS for both PVC and LDPE as well as minimal adsorption of MC-LR and -RR for all polymer types, likely due to the formation of oxygen-containing surface groups and associated reduction in hydrophobicity when compared to virgin particles. Virgin plastic materials are typically hydrophobic, which allows them to more readily adsorb organic contaminants in water. These findings suggest that microplastics in the environment, which undergo natural weathering, may adsorb lower amounts of PFAS and microcystins than previously anticipated based on studies that employed only virgin polymers.

Nonetheless, adsorption should be considered in the evaluation of risks to human health via consumption of drinking water. Future studies should ideally examine smaller microplastics (<20 µm) that are naturally weathered in order to confirm adsorption behavior.

## Figures and Tables

**Table 1:** Mean surface roughness of virgin and weathered polymers (*n* = 10).

Polymer Type	Mean Roughness (µm)		*p* Value
	Virgin	Weathered	
LDPE	0.74 ± 0.59	0.47 ± 0.61	0.36
PET	0.78 ± 0.55	0.99 ± 0.89	0.56
PS	0.49 ± 0.47	0.19 ± 0.10	0.07
PMMA	0.32 ± 0.28	0.18 ± 0.11	0.19
PVC	1.23 ± 0.67	1.64 ± 2.21	0.60

## Data Availability

Data will be made available upon request.

## Supplementary Materials

The following supporting information can be downloaded at: https://www.mdpi.com/article/10.3390/polym15183676/s1.
