# Supplementary material for: Adsorption of Per- and Polyfluoroalkyl Substances (PFAS) and Microcystins by Virgin and Weathered Microplastics in Freshwater Matrices
Source: Polymers (Basel). 2023 Sep 6;15(18):3676. doi: 10.3390/polym15183676 (PMC10535594; doi:10.3390/polym15183676)
Supplement: Supplementary file 1 [file polymers-15-03676-s001.zip › polymers-2564734-supplementary.pdf]

Supplementary Information
For

Adsorption of Per- and Polyfluoroalkyl Substances (PFAS) and Microcystins by Virgin and Weathered Microplastics in Freshwater Matrices

Yucong Shi, Husein Almuhtaram*, and Robert C. Andrews

Department of Civil and Mineral Engineering, University of Toronto, 35 St. George St., Toronto, Ontario, M5S 1A4, Canada

*Corresponding author: Husein Almuhtaram (husein.almuhtaram@utoronto.ca)

Mailing address:
Department of Civil and Mineral Engineering
35 St. George Street
Toronto ON M5S 1A4
Canada

Results from initial PFAS isotherm and kinetic trials using virgin 200 and 1090 µm PE

Initial isotherm trials examined the adsorption of four PFAS compounds individually at 500 ng/L by 900-3600 mg/L of 200 and 1090 µm PE in AFW. Following a contact period of 21 d, residual concentrations for short-chain PFBA and PFBS were very close to the control level, indicating little to no adsorption by virgin PE microplastics (Fig. S3). However, when considering long-chain PFOA and PFOS, a decrease in concentration was observed, especially for 200 µm PE, suggesting that adsorption increases with surface area since the specific surface area of 200 µm particles is approximately 40 times greater than that of 1090 µm particles. Udenby et al. (2022) reported adsorption of fluoranthene and phenanthrene by 200 µm PE particles to increase by factors of 1.2 and 1.5, respectively, when compared to adsorption by 1090 µm PE particles. Here, the average adsorption of PFOA and PFOS by the smaller particles increased by factors of 2.7 and 4.7, respectively.

It is not surprising that low absorption was observed when considering 500 ng/L as an initial PFAS concentration. Previous studies that examined microplastic adsorption of PFAS compounds (Llorca et al., 2018) and PAHs (Udenby et al., 2022; Wang and Wang, 2018), used considerably higher contaminant concentrations. For example, Llorca et al. (2018) spiked 18 PFAS compounds at 10 µg/L (20 times the concentration used here) as a mixture into freshwater and seawater matrices, as well, 3-16 µm high density PE was used, (at least 12 times smaller than particles in this study).

Table S1. Summary of weathering methods related to microplastics and their impact on adsorption.

Water Matrix	Adsorbate	Polymer Type	Polymer Size	Weathering Method	Surface characteristics associated with weathering	Impact of Weathering on Adsorption	Reference
Ultrapure water	PAHs and Antibiotics	PS	1 µm	75 °C in air, freshwater, and seawater in petri dishes	FTIR: increased oxygen-containing surface groups	Enhanced adsorption of antibiotics but decreased adsorption of PAHs	Ding et al. (2020)
Ultrapure water and lake water	Microcystins	LDPE, PET, PS, PVC	3-5 mm	Naturally weathered in lake water	Not reported	Decreased adsorption in lab trials but enhanced adsorption in in-situ experiments	Hataley (2020)
Freshwater and simulated seawater	Antibiotics	PS, PE	0.5-2 mm	Naturally weathered in sea water	FTIR: increased oxygen-containing surface groups	Only weathered particles included	Guo and Wang (2019)
Ultrapure water	Cu^{2+}	PA, PE, PS, PET, PVC, PMMA	70-350 µm	Weathered under UVA-340 nm	FTIR: decreased carbonyl index (CI) of PA, increased CI for PMMA	Increased adsorption for PMMA but decreased adsorption for PA	Yang et al. (2019)

Water Matrix	Adsorbate	Polymer Type	Polymer Size	Weathering Method	Surface characteristics associated with weathering	Impact of Weathering on Adsorption	Reference
Freshwater	Strong (Cs) and weak (Sr) cations	HDPE, PP	50-100 µm	Preconditioned with gamma-irradiation and incubated in freshwater and sea water.	FTIR: increased oxygen-containing surface groups	Only weathered particles included	Johansen et al. (2019)
Ultrapure water	Antibiotics	HDPE, PS	50 µm	Heat-activated $K_2S_2O_8$ treatment and Fenton treatment	SEM: formation of cracks and pits, fragmentation of particles;<br>XPS: increased O/C ratio<br>Contact Angle: decreased surface hydrophobicity<br>FTIR: increased CI for both polymers	Enhanced adsorption of antibiotics	Liu et al. (2019)
Freshwater	N/A	PET, PVC	Not reported	Naturally weathered in river water	SEM: increased surface roughness<br>XPS: increased O/C ratio<br>FTIR: increased oxygen-containing surface groups	N/A	Dong et al. (2020)

Water Matrix	Adsorbate	Polymer Type	Polymer Size	Weathering Method	Surface characteristics associated with weathering	Impact of Weathering on Adsorption	Reference
Sea water	Endocrine disrupters	PE	< 1 mm	Naturally weathered in sea water	Not reported	Increased genotoxicity on fish, suggesting increased adsorption	Rochman et al. (2014)
Sea water	PAHs	PS, PET, LDPE, HDPE, PVC, PP	3 mm	Naturally weathered in sea water	Not reported	Enhanced adsorption of PAHs until equilibrium	Rochman et al. (2013)
Freshwater	Bisphenols and parabens	PE, PVC, PA	90-228 µm	Immersed in algal bioreactor and river freshwater	SEM: formation of cracks, pits, and wrinkles, and increased roughness<br>Size Distribution increased surface area<br>Contact Angle: decreased surface hydrophobicity<br>FTIR: increased oxygen-containing surface groups	Increased adsorption capacity of bisphenols and parabens by 2-9 times.	Kiki et al. (2022)

Water Matrix	Adsorbate	Polymer Type	Polymer Size	Weathering Method	Surface characteristics associated with weathering	Impact of Weathering on Adsorption	Reference
Sea water	Antibiotics	PS	0.45-1 mm	Naturally weathered on coastal beaches	SEM: formation of cracks, pits, and wrinkles, and increased roughness<br>FTIR: increased oxygen-containing surface groups	Enhanced adsorption of antibiotics	Zhang et at. (2018)

References

Adeleke, O.A., Latiff, A.A.A., Saphira, M.R., Daud, Z., Ismail, N., Ahsan, A., Ab Aziz, N.A., Al-Gheethi, A., Kumar, V., Fadilat, A., Apandi, N., 2019. 1 - Principles and Mechanism of Adsorption for the Effective Treatment of Palm Oil Mill Effluent for Water Reuse, in: Ahsan, A., Ismail, A.F. (Eds.), Nanotechnology in Water and Wastewater Treatment, Micro and Nano Technologies. Elsevier, pp. 1–33. https://doi.org/10.1016/B978-0-12-813902-8.00001-0

Chen, C.C., Zhu, X., Xu, H., Chen, F., Ma, J., Pan, K., 2021. Copper Adsorption to Microplastics and Natural Particles in Seawater: A Comparison of Kinetics, Isotherms, and Bioavailability. Environ. Sci. Technol. 55, 13923–13931. https://doi.org/10.1021/acs.est.1c04278

Ding, L., Mao, R., Ma, S., Guo, X., Zhu, L., 2020. High temperature depended on the ageing mechanism of microplastics under different environmental conditions and its effect on the distribution of organic pollutants. Water Res. 174, 115634. https://doi.org/10.1016/j.watres.2020.115634

Dong, M., Zhang, Q., Xing, X., Chen, W., She, Z., Luo, Z., 2020. Raman spectra and surface changes of microplastics weathered under natural environments. Sci. Total Environ. 739, 139990. https://doi.org/10.1016/j.scitotenv.2020.139990

Du, Z., Deng, S., Bei, Y., Huang, Q., Wang, B., Huang, J., Yu, G., 2014. Adsorption behavior and mechanism of perfluorinated compounds on various adsorbents—A review. J. Hazard. Mater. 274, 443–454. https://doi.org/10.1016/j.jhazmat.2014.04.038

Guo, X., Wang, J., 2019. The phenomenological mass transfer kinetics model for Sr2+ sorption onto spheroids primary microplastics. Environ. Pollut. 250, 737–745. https://doi.org/10.1016/j.envpol.2019.04.091

Hataley, E.K., 2020. Sorption of the Common Freshwater Cyanotoxin Microcystin to Microplastics. Queen’s University.

Johansen, M.P., Cresswell, T., Davis, J., Howard, D.L., Howell, N.R., Prentice, E., 2019. Biofilm-enhanced adsorption of strong and weak cations onto different microplastic sample types: Use of spectroscopy, microscopy and radiotracer methods. Water Res. 158, 392–400. https://doi.org/10.1016/j.watres.2019.04.029

Kiki, C., Qiu, Y., Wang, Q., Ifon, B.E., Qin, D., Chabi, K., Yu, C.-P., Zhu, Y.-G., Sun, Q., 2022. Induced aging, structural change, and adsorption behavior modifications of microplastics by microalgae. Environ. Int. 166, 107382. https://doi.org/10.1016/j.envint.2022.107382

Liu, P., Qian, L., Wang, H., Zhan, X., Lu, K., Gu, C., Gao, S., 2019. New Insights into the Aging Behavior of Microplastics Accelerated by Advanced Oxidation Processes. Environ. Sci. Technol. 53, 3579–3588. https://doi.org/10.1021/acs.est.9b00493

Llorca, M., Schirinzi, G., Martínez, M., Barceló, D., Farré, M., 2018. Adsorption of perfluoroalkyl substances on microplastics under environmental conditions. Environ. Pollut. 235, 680–691. https://doi.org/10.1016/j.envpol.2017.12.075

Mohammadi, M.M., 2015. The bioavailability of perfluoroalkyl substances (PFASs) and polycyclic aromatic hydrocarbons (PAHs) in soil to Eisenia fetida and Cucurbita pepo 51.

Pivokonsky, M., Cermakova, L., Novotna, K., Peer, P., Cajthaml, T., Janda, V., 2018. Occurrence of microplastics in raw and treated drinking water. Sci. Total Environ. 643, 1644–1651. https://doi.org/10.1016/j.scitotenv.2018.08.102

Rochman, C.M., Hoh, E., Hentschel, B.T., Kaye, S., 2013. Long-Term Field Measurement of Sorption of Organic Contaminants to Five Types of Plastic Pellets: Implications for Plastic Marine Debris. Environ. Sci. Technol. 47, 1646–1654. https://doi.org/10.1021/es303700s

Rochman, C.M., Hentschel, B.T., Teh, S.J., 2014. Long-Term Sorption of Metals Is Similar among Plastic Types: Implications for Plastic Debris in Aquatic Environments. PLoS One 9, e85433. https://doi.org/10.1371/journal.pone.0085433

Subramanyam, B., Das, A., 2009. Linearized and non-linearized isotherm models comparative study on adsorption of aqueous phenol solution in soil. Int. J. Environ. Sci. Technol. 6, 633–640. https://doi.org/10.1007/BF03326104

Sun, Y., Yuan, J., Zhou, T., Zhao, Y., Yu, F., Ma, J., 2020. Laboratory simulation of microplastics weathering and its adsorption behaviors in an aqueous environment: A systematic review. Environ. Pollut. 265, 114864. https://doi.org/10.1016/j.envpol.2020.114864

Udenby, F.A.O., Almuhtaram, H., McKie, M.J., Andrews, R.C., 2022. Adsorption of fluoranthene and phenanthrene by virgin and weathered polyethylene microplastics in freshwaters. Chemosphere 135585. https://doi.org/10.1016/j.chemosphere.2022.135585

Wagstaff, A., Lawton, L.A., Petrie, B., 2022. Polyamide microplastics in wastewater as vectors of cationic pharmaceutical drugs. Chemosphere 288, 132578. https://doi.org/10.1016/j.chemosphere.2021.132578

Wang, W., Wang, J., 2018. Comparative evaluation of sorption kinetics and isotherms of pyrene onto microplastics. Chemosphere 193, 567–573. https://doi.org/10.1016/j.chemosphere.2017.11.078

Yang, J., Cang, L., Sun, Q., Dong, G., Ata-Ul-Karim, S.T., Zhou, D., 2019. Effects of soil environmental factors and UV aging on Cu2+ adsorption on microplastics. Environ. Sci. Pollut. Res. 26, 23027–23036. https://doi.org/10.1007/s11356-019-05643-8

Zhang, H., Wang, J., Zhou, B., Zhou, Y., Dai, Z., Zhou, Q., Chriestie, P., Luo, Y., 2018. Enhanced adsorption of oxytetracycline to weathered microplastic polystyrene: Kinetics, isotherms and influencing factors. Environ. Pollut. 243, 1550–1557. https://doi.org/10.1016/j.envpol.2018.09.122
